# The Use of Intersectional Analysis in Assessing Women’s Leadership Progress in the Health Workforce in LMICs: A Review

**DOI:** 10.34172/ijhpm.2021.06

**Published:** 2021-02-09

**Authors:** Zahra Zeinali, Kui Muraya, Sassy Molyneux, Rosemary Morgan

**Affiliations:** 1 Department of International Health, Johns Hopkins Bloomberg School of Public Health, Baltimore, MD, USA; 2 Kenya Medical Research Institute (KEMRI) - Wellcome Trust Research Programme, Nairobi, Kenya; 3 Nuffield Department of Medicine, Oxford University, Oxford, UK

**Keywords:** Gender, Intersectionality, Health Systems, Health Workforce, Leadership, Low- and Middle-Income Countries (LMICs)

## Abstract

**Background:**

Human resources are at the heart of health systems, playing a central role in their functionality globally. It is estimated that up to 70% of the health workforce are women, however, this pattern is not reflected in the leadership of health systems where women are under-represented.

**Methods:**

This systematized review explored the existing literature around women’s progress towards leadership in the health sector in low- and middle-income countries (LMICs) which has used intersectional analysis.

**Results:**

While there are studies that have looked at the inequities and barriers women face in progressing towards leadership positions in health systems within LMICs, none explicitly used an intersectionality framework in their approach. These studies did nevertheless show recurring barriers to health systems leadership created at the intersection of gender and social identities such as professional cadre, race/ethnicity, financial status, and culture. These barriers limit women’s access to resources that improve career development, including mentorship and sponsorship opportunities, reduce value, recognition and respect at work for women, and increase the likelihood of women to take on dual burdens of professional work and childcare and domestic work, and, create biased views about effectiveness of men and women’s leadership styles. An intersectional lens helps to better understand how gender intersects with other social identities which results in upholding these persisting barriers to career progression and leadership.

**Conclusion:**

As efforts to reduce gender inequity in health systems are gaining momentum, it is important to look beyond gender and take into account other intersecting social identities that create unique positionalities of privilege and/or disadvantage. This approach should be adopted across a diverse range of health systems programs and policies in an effort to strengthen gender equity in health and specifically human resources for health (HRH), and improve health system governance, functioning and outcomes.

## Background

It is estimated that globally, the health sector has the highest proportion of women in the workforce compared to other sectors.^1^ According to the World Health Organization (WHO), in many countries, up to 70% of the health workforce are women.^2^ In pre-service education, including in medical schools and other healthcare related fields of academia, women’s representation has increased, achieving near-parity.^3^ Nonetheless, leadership in the health sector remains dominated by men, including in top global institutions and multilateral organizations, governments, private sector, and in decision-making structures. Women remain occupying the lower hierarchy positions such as nurses, midwives and community health workers, cadres that often represent positions of lesser authority (but not importance) across the health sector.^4,5^ For example, in 2020 only 3.5% of the 115 identified national coronavirus disease 2019 (COVID-19) task forces had gender parity among members and only 25% of ministers of health were women; at the 72nd World Health Assembly in 2019 only 22% of member state delegations had a woman as a chief delegate, down from 29% in the previous years; in 2016 only 33.5% of WHO Country Office heads were women.^4,6–9^ The Global Health 50/50 reports released since 2018, aimed to shed some light on gender equality in 200 major organizations working in and/or influencing the field of global health. The reports note that decision-making power remains in the hands of men, comprising 68% and 80% of board chairs and 73% and 69% of executive directors in the organizations that were examined in 2020 and 2018, respectively.^10–12^

According to the WHO, gender is a social construct that refers to the characteristics of men and women, including norms, behaviors, expectations and roles associated with being a man or a woman. The concept of gender varies in different societies and over time. Gender interacts with but is different from sex, which refers to the biological attributes of males, females and intersex people.^13^ For the purpose of this study we use the words woman/women and man/men when referring to gender instead of the more familiar but inaccurate terms female and male.

In a gender analysis of human resources for health (HRH) for the WHO, George notes that *“gender, among other power relations, plays a critical role in determining the structural location of women and men in the health labor force and their subjective experience of that location”* (p. 5).^14^ The under-representation of women in leadership positions across health systems, the pay-gap, and physical and sexual violence and harassment are all rooted in gender biases in norms, power, access to resources and entitlements, and values.^1,15^ The Lancet series on Gender Equality, Norms, and Health further emphasizes that health systems contribute to gender inequalities in health by replicating and reinforcing restrictive gender norms and gender inequalities.^5,16^ It is for this reason that we focused this study on women’s representation in leadership, while acknowledging and emphasizing that gender inequities go beyond the inequities women face. In order to avoid making assumptions and blanket recommendations, it is necessary to examine how gender intersects with other social identifiers (such as age and race) and social stratifiers (such as socio-economic status and professional cadre), and how these are embedded within broader structures of power to influence the professional advancement of women. An intersectional approach allows us to do this.

Intersectionality, a concept first introduced by Kimberlé Crenshaw three decades ago, has emerged as a framework for explaining and addressing inequities, and more recently gaining popularity in health outcomes and systems research.^17^ Intersectionality is defined as promoting “*an understanding of human beings as shaped by the interaction of different social locations such as race/ethnicity, indigeneity, gender, class, sexuality, geography, age, disability/ability, migration status, religion. These interactions occur within a context of connected systems and structures of power including laws, policies, state governments and other political and economic unions, religious institutions and media. Through such processes, interdependent forms of privilege and oppression shaped by colonialism, imperialism, racism, homophobia, ableism and patriarchy are created”* (p. 2).^18^

Examples of how inequity manifests in HRH are available in the literature: studies conducted in South Africa have called for a need for improved racial and gender diversity among healthcare providers to meet the needs of diverse societies such as South Africa.^19,20^ Assessments of cadre inequality in leadership positions in various settings have shone a light on the over-representation of certain healthcare professional categories, such as physicians, that are traditionally considered more “elite.”^4,21^ These so-called elite cadres are historically dominated by men, which further exacerbates gender inequity in health leadership. This gender imbalance in certain professional cadres (eg, nursing being more dominated by women and medicine, dominated by men) is referred to as horizontal occupational gender segregation, a phenomenon known to contribute to lack of motivation and low morale, disempowerment, and maldistribution of the workforce.^22^

Improving gender equity in health systems and institutions allows for building more equitable and just health systems, unleashing the full potential of the workforce that ultimately benefits populations and their health outcomes. While gender is crucial to understanding opportunities, power and privilege, an intersectional approach allows for a more nuanced view of the dynamics currently at play and levers to sustainably improve health workforce leadership. To this end, the Third Global Forum on HRH produced the Recife Political Declaration on HRH which committed to “*promote equal opportunities in education, development, management and career advancement for all health workers, with no form of discrimination based on gender, race, ethnicity or any other basis”* (p. 3).^23^ This commitment displays the growing recognition of the role of gender and intersectional approaches.

This review examines the literature around leadership in the health sector in low- and middle-income countries (LMICs), with an explicit intersectionality lens.

## Methods

We conducted a systematized review of the literature on intersectionality in health systems leadership in the context of LMICs. A systematized review process models the systematic review process but lacks some features of a full systematic review, such as quality assessment of identified literature or two reviewers.^24^ We selected this approach because of the need for more flexibility in the review process, recognizing that there would likely be minimal articles published on this topic.

Literature for the systematized review was identified using five electronic databases; PubMed, CINAHL, Embase, Scopus, and Web of Science. The search keywords and concepts included ‘leadership,’ ‘gender,’ ‘human resources for health’ and ‘health systems,’ ‘intersectionality’ and ‘low and middle-income countries.’ The search terms included relevant equivalents for each concept according to the databases. A combination of search terms was used within each database, using the Boolean operators AND, OR. The search was initially conducted in April 2018 and updated in October 2020 to cover any published papers between April 2018 and October 2020, and included articles starting from January 2000. We limited the search to articles in English. Titles and abstracts were included if they met all inclusion criteria as described in Table 1. After removing duplicates, the electronic search yielded a total of 7709 titles/abstracts, which were further screened, using the key words and concepts of interest, resulting in 48 articles (see PRISMA [Preferred Reporting Items for Systematic Reviews and Meta-Analyses] flow chart in Figure). Fourteen of these 48 articles were selected as relevant to gender and leadership in health systems. Upon closer examination of the full text of each article, applying the inclusion criteria, no articles were found that had used an intersectionality framework to explore health system leadership in LMICs. While the 14 articles did not include a discussion about intersectionality as an approach, perspective, framework, or lens, they each did discuss the role of multiple social stratifiers and experiences of health systems leadership. We therefore included the 14 articles, and on reviewing those papers added a further five studies they cited, increasing the number of included papers to 19. An additional seven articles were included based on the recommendation of experts in the field, identified based on their publications, resulting in a total of 25 articles. None of the final 25 papers included an explicit intersectionality lens, but all touched on arguments relevant to this study (Figure, PRISMA flow chart).

Each of the final 25 articles was examined using an intersectional lens. This was done through coding any social stratifiers mentioned in relation to barriers in progressing towards leadership positions. If the social stratifier had been mentioned in the context of gender or there was a mention of how this stratifier intersects with gender, this was also specifically coded and included in Table 2.^4,5,14,16,17,19–22,25–40^ Similar codes were grouped together and gave rise to the emerging themes that have been included in the findings section of this study.

## Results

### Gender and Leadership in Health Systems

In the initial steps of the search strategy, we found that the intersectionality framework has mainly been used in the context of social determinants of health and understanding barriers to accessing healthcare in different settings. Much of the literature focusing on women’s participation in the health sector focused on the broad categories of health professions rather than more specifically on leadership within those professions. In order to understand how gender intersects with other social stratifiers to influence women’s experiences of and opportunities for leadership it is important to first explore the role of gender and how gender inequity manifests within health systems leadership structures. The gender-related barriers identified in this review were focused on barriers women face and included (1) women’s relative lack of access to resources that improve career development, (2) women’s relative lack of access to mentorship and sponsorship opportunities, (3) lack of value, recognition and respect at work for women and the attribution of success to feminine traits rather than professional competence, expertise or hard work, (4) greater likelihood by women to take on dual burdens of professional work and childcare and domestic work, and (5) assumptions that women have leadership styles that are less effective for top management compared to men. It is notable that there was a dearth of discussion of how these barriers might manifest differently for women from different demographic groups including racial categories and professional cadre, which have been highlighted in literature as resulting in differential experiences for health workers.^25,26^ Nonetheless, other social stratifiers were discussed to a limited extent within the studies, and these are further explored below. Table 2 summarizes the emerging themes in the reviewed studies.

When exploring leadership as a professional concept and related gendered experiences, Tominc et al highlight that within a broad pattern of women getting fewer leadership opportunities than men, there is also an evolving recognition of a phenomenon of women being offered particularly challenging opportunities, referred to as “*glass cliff*” opportunities. This term is used to describe a situation where women are offered leadership positions when uncertain and risky conditions decrease the likelihood of their success and set them up them for failure and poor outcomes (eg, fasttrack promotion of women to leadership in failing or near bankruptcy companies).^27^ This, in turn, can have adverse implications for external views about women’s abilities as leaders and managers, as well as women’s own internal view about their abilities, potentially discouraging them from taking up leadership positions.^27^

Gendered societal norms and expectations also have a significant influence on women’s interest in, and ability to, participate in health leadership.^28^ A study in Cambodia demonstrated that managers who were men emphasized that women’s roles and priorities should be centered around their household responsibilities.^29^ Similarly, a study conducted with mid-level health managers in Kenya found that women were perceived as having primary responsibility for child nurturing which in turn impacts their willingness and ability to take up senior health management positions, and hinders their career progression.^25^ Another study with health system managers in South Africa described a manager who was a woman who aspired to be a surgeon alongside her husband, but decided to “*put her career on the back burner*” when they had children and “*allow her husband to follow his dreams*.”^26^

A study on women in different professions (outside of the health system) in Sri Lanka, noted that the current work norms of working long hours, stress, and competition, promote a “*masculinization of management*.”^30^ The general perception being that for women to be accepted as leaders they need to act like men, but in doing so they risk losing their obligatory attributes of femininity. The study noted that if women try to take on leadership roles building on their feminine attributes, it would be viewed as challenging the right of men to hold positions of power. At the same time, women leaders using those same feminine attributes were at risk of being undermined for an apparent incapability to do the job.^30^ The description of said feminine attributes was not elaborated on in the study.

Lastly, Zeinali et al have called for incorporating intersectionality in health workforce policy and planning, to account for structures of power beyond the health system that affect woman health workers’ career progression. These intersectional considerations are time and location specific and therefore need strong political will, commitment and transparency to be incorporated in health systems and allow for meaningful, non-tokenistic and meritocratic inclusion of health workers at all leadership levels.^31^

#### Gender and Childcare/Motherhood: Gendered Roles and Responsibilities

Five studies, conducted by George,^14^ Newman,^22^ Alwazzan and Rees,^32^ Ioannidou et al^33^ and Muraya et al^25^ mentioned gender and childcare/motherhood in the context of gendered roles and responsibilities.

In a gender analysis of the HRH done for the WHO, George points to stereotypical work models that assume men are the default and ignore the differences women have, expecting them to conform to the default, or associate these differences to individual women rather than differences structured by the social environment. One such major difference is in the domestic and childcare responsibilities women take up.^14^ Most workplaces situated in the health system do not account for the specific conditions and needs that exist for woman health workers who have children or are in charge of a household, complicating both their professional and personal lives.^34^

In a commentary on gender inequality in the health workforce, Newman points to the discrimination women face in recruitment and promotion processes due to the possibility of pregnancy. This discrimination also manifests as lack of organizational support to mothers such as flexible work conditions, parental leave or childcare.^22^

Muraya et al examined the role of parenthood, family support and domestic help in the professional experience of healthcare managers in Kenya. The topic of receiving support from family and spouses came up with both men and women study participants, however women tended to emphasize more on the positive effects of having a supportive family who appreciate the time constraints of their work. The demands of parenthood came up mainly in the interviews with women healthcare managers, however, it was used as a justified reason for exclusion of women from leadership positions by both men and women in the study.^25^

Similarly, Alwazzan and Rees pointed to cultural norms and expectations for women in Saudi Arabia, highlighting that society, families, and employers all expect women to prioritize childbearing and childcare. Many of the women participating in the study agreed with prioritizing motherhood, but pointed to the significant role that families, spouses and workplaces can have in creating a supportive and enabling environment. Women in this study were healthcare academics and emphasized the role of organizational support that allows for more flexible work conditions conducive to childcare, as well as elimination of biases that leads to women being overlooked for leadership positions.^32^

In their analysis of the dental workforce, Ioannidou et al explore various forms of gender discrimination in this cadre and the barriers women face, including the double burden of professional work and childcare. Lack of institutional support for childcare, in terms of allocation of dedicated space and caregivers, financial support and considerations for tenure have all been mentioned to hinder women’s advancement to leadership positions in the dental workforce and academia.^33^

### Intersection of Gender With Other Social Stratifiers

#### Gender, Professional Cadre and Race

Eight studies addressed the intersection of gender, professional cadre and race: Muraya et al,^25^ Shung-King et al,^26^ Dhatt et al,^4^ Filby et al,^21^ Thackwell et al,^19^ van Rensburg,^20^ Williams,^35^ and George.^36^

Muraya et al and Dhatt et al report that in Kenya, professional hierarchies play an important role in the appointment of health leaders, with medical doctors being preferentially appointed into leadership positions.^4,25^ This in and of itself is gendered as the medical field in Kenya was historically dominated by men, although this has gradually changed over time with equal numbers of men and women entering into medical school and holding entry-level medical positions. This is an example of how gender and professional cadre can intersect to influence women’s participation as leaders in the health system.

George discussed that in Iran, nurses, which is a cadre dominated by women, were reported to avoid being the decision-makers in a healthcare team even when they had the knowledge and skills, because their autonomy and authority was undermined by the physician-centered culture of the health systems they worked in.^36^ The healthcare culture was found to revolve around physicians. Teamwork with other cadres was less valued, leading to the discouragement and disempowerment of other professional cadres in taking on leadership roles in health teams. This illustrates how professional cadre can intersect with gender and work cultures, limiting the participation of women in leadership spaces, even in professions dominated by women such as nursing and midwifery as further elaborated below.

A study by Filby et al explored the intersectional nature of gender and professional cadre within midwifery leadership in LMICs. They examined the concept of “*gender penalty*” to describe the phenomenon they observed where men assume leadership positions even in professions that are disproportionately comprised of women, such as nursing and midwifery, leaving women to fill the bottom of the occupational hierarchy. This, in part, is due to women’s job-related skills in caring professions not being treated as professional skills, but rather as qualities of being a woman.^21^

Another way in which gender intersects with professional cadre is what Williams describes as the “*glass escalator*.” The glass escalator refers to the advantages that men receive even in professions dominated by women such as nursing, allowing them to climb towards leadership levels more easily and quickly compared to women.^34^ This is in contrast to the previously discussed “*glass cliff*” concept, where women are put in leadership positions with low success rates, essentially positioning them for likely failure.^27^

Nonetheless, the “escalator” advantage may not necessarily privilege all men equally, as prior research has shown that behaviors that denote leadership ability in White men are perceived as menacing behavior from Black men,^35^ highlighting the importance of an intersectional approach. As such, gender intersects with race in this case to disadvantage Black men and potentially prevent them from exercising their leadership traits.^35^ This example gives additional context to the complex nature of systems of oppression that intersect to create unique experiences of disadvantage. In the South African study of health systems managers’ experiences, Shung-King et al observed a combination of gender, race and professional hierarchy in influencing leadership experience. To illustrate, they described a Black manager who was a man recalling that (in addition to the prejudice he experienced as a Black man), as a nurse who was a man he had often faced prejudice from his nurse colleagues who were women, as well as family members and social circles for doing “a woman’s job.”^26^ Although his experience did not follow the usual pathways of structural discrimination, it was drawn upon to show how strongly gender intersects with other social identities in influencing people’s experiences. The same study found that Black woman managers from a nursing background, experience a “*triple-challenge*” of gender, professional hierarchy and race in their professional life, even post-apartheid. The authors argue that although in theory equal rights now exist for all regardless of race in South Africa, other insidious forms of discrimination still persist, resulting in unique experiences for health professionals based on the intersections of their gender, race and professional hierarchies. In the other South African focused study, van Rensburg confirms this theory and discusses how the skewed race and gender profiles of the health workforce still persist to some degree, despite affirmative action policies in place.^20^ When exploring affirmative action policies in South Africa for the training of medical specialists, Thackwell et al discussed the concept of “*race fatigue*” where Black trainees felt dissatisfied with being the token Black trainee in a White-dominated workplace. Moreover, Black study participants mentioned hostile attitudes towards them due to biases that considered them less competent and ignored their merit because of the affirmative policies. This attitude created implicit barriers in their career progression in the medical field.^19^

Another example of the complex effects of the intersection of gender with race is highlighted by George in her 2008 study in the United States. She discusses how government funding assists minority women to be trained for lower levels of nursing, leading to their prominent presence at this level. However, the funding does not apply to baccalaureate training which determines teaching and leadership positions, resulting in an inevitable shortage of minority women in higher-level and decision-making spaces.^36^ This also highlights how well-intentioned policies and interventions – in this case government-assisted education funding – can inadvertently disadvantage intended beneficiaries, “locking” them into particular lower-level categories and limiting their career progression into leadership roles.

#### Gender, Race, and Ethnicity

Two of the reviewed studies – one in Singapore,^37^ the other in South Africa^20^ – examined the intersection of gender and race/ethnicity. The Singapore study focused on the disadvantages men experience in professional settings outside of the health system, but the complex nature of how systems of oppression intersect to create experiences of disadvantage made it important to include in this review to provide additional context. The study examines social capital and the intersection of gender and ethnicities. The authors found that while men of all major ethnicities living in Singapore have an advantage accessing university education compared to their woman counterparts, there is one exception: Malay men are at a disadvantage compared to Malay women when it comes to access to university education. The authors elaborate that this disadvantage experienced by Malay men leads to lower social capital over time, for instance knowing fewer people in high-status jobs. Therefore, while men in general seem to be at an advantage for higher education in Singapore, Malay men experience a unique disadvantage due to the intersection of their ethnicity with their gender.^37^

In the South African study, van Rensburg examines the distorted race and gender profiles of the health workforce that persist despite the progress made by affirmative action policies in the post-apartheid government. van Rensburg argues that remnants of historical exclusion based on race including in the higher education sector and white-collar professions continue to exist to date in South Africa. This is for example observed in the number of medical practitioners nationally: 16936 (Whites), 8354 (African Blacks), 5314 (Indian) and 927 (colored) (labels are used verbatim from the original paper).^20^ This is in a country where Black Africans make up 80.2% of the population, with colored, White and Indian/Asian making up 8.8%, 8.4% and 2.5% of the population respectively.^41^ The outcomes of this racial exclusion, marked by white privilege and dominance, are further skewed by the dominance of men.^20^

#### Gender and Culture

Two studies examined gender and culture. Tlaiss^34^ looked at gender and religion/religious culture and Alwazzan and Rees^32^ studied gender and generation/age. The study discussing gender and religion in Lebanon’s health sector noted that 79% of the public health workforce is comprised of women, but this is not reflected in the leadership positions. Tlaiss considered socio-cultural factors and Lebanon’s society that reinforces traditional gender roles around domestic responsibilities as the key reasons hindering women’s upward movement in the hierarchy of the health system.^34^ Furthermore, she discusses the role of religion even in this broader context of gendered norms and roles; highlighting that in Lebanon, Muslim communities are generally more conservative and traditional than Christian communities leading to an underrepresentation of Muslim women in the workforce as a result of traditional norms and obligations placing a higher value on motherhood than social and economic participation. Tlaiss also highlighted the patriarchal organizational culture and implicit biases that see and appoint men as default managers and leaders.^34^

Alwazzan and Rees conducted their study in Saudi Arabia with women faculty of medical colleges and explored gender and generation.^32^ They noted that both implicit (for example gender stereotyping) and explicit barriers (such as lack of research opportunities, lack of mentorship, and difficulty in achieving work-life balance) were identified by participants as hindering their career progression. Some examples of gender stereotypes that were given by the study participants included women lacking the physical strength for some medical specialties, or lacking personality traits that would make them *“fit for leadership positions.”*^32^ The study discussed the intersecting axes of gender and culture when participants stated that their culture places a higher value on domestic responsibilities for women rather than working professionally and is less encouraging of women to occupy professional spaces. Furthermore, women face generational barriers, in that young professionals (of any gender) are not taken seriously despite being qualified, and leadership positions are more readily available to older individuals, even when individuals are equally qualified for a leadership position.

#### Gender and Financial Status

The study done by Muraya et al looked at the intersection of gender and financial status. In this study, healthcare managers in Kenya nodded to different factors that have enabled or hindered their progress along their careers, emphasizing the importance of financial support in enabling them to take further education or training to better prepare them for leadership. Women also pointed to competing financial interests such as family obligations or prioritizing the cost of their children studying.^25^ Many of the participants in this study who had children, also emphasized the importance of having domestic help for their household responsibilities, most of which are often expected of women.^25^ Affording domestic help is another manifestation of the intersection of gender and financial status.

## Discussion

We explored the literature around intersectionality of gender and other social axes in health systems leadership, with a focus on LMICs. None of the articles identified using our search strategy had explicitly used intersectional analysis to assess how gender intersects with other social stratifiers. Despite this, it was clear from the 25 papers we did include that gender intersects with other social stratifiers in unique ways to influence experiences and career progression of health systems leaders.

There has been an upturn of focus on the role of women in health systems, and in particular in leadership positions, such as the WHO Global Strategy on Human Resources for Health: Workforce 2030,^1^ UN High Level Commission on Health Employment and Economic Growth,^42^ the Global Health 50/50 reports,^10–12^ The Lancet special theme issue on Advancing Women in Science, Medicine, and Global Health,^43^ The Lancet series on Gender Equality, Norms, and Health,^44^ and initiatives such as Women in Global Health and the Gender Equity Hub of the Global Health Workforce Network,^45^ and most recently, calls for women leading the post-COVID-19 pandemic efforts due to their exemplary leadership of the pandemic response.^9,46,47^

However, most of these efforts fall short in that they focus mainly on homogenously increasing women’s participation in leadership, with little attention is paid to other social axes that intersect with gender to inhibit progression to higher-level positions. This linear, single-layered approach does not reflect the complexities of real-world experiences, including systems and structures of power that interact to privilege certain women over others while excluding many groups of competent women who are minoritized beyond their gender.

An intersectionality approach explicitly focuses on the relationships between mutually constructed processes, systems and structures that lead to social differences and inequities.^18^ Acknowledging the dynamic interconnectedness of gender with other social identities and locations, especially when considering women who do not fulfill the often portrayed and represented description of women in leadership, avoids delays in adopting solutions that benefit women from different backgrounds and lived experiences. A failure to recognize and analyze diversity among women in leadership risks the portrayal of women leaders as primarily White and from/in high income countries, and of under-representing the visibility and voice of other women leaders. By that very fact, in order to lay a strong, inclusive foundation for change in the gender equality agenda, there have been calls for more visible leadership in global health from LMIC feminist voices.^48^ Furthermore, leadership concepts and practices are deeply connected to one’s identity and lived experiences. Incorporating intersectional analysis in the study of leadership allows for having a full view of one’s identity and approach to leadership.

Health systems are often viewed as gender neutral, technical systems, but in reality, they are complex systems imbued with power relations. They are embedded in and shaped by their socio-political contexts, thereby reflecting and reinforcing social norms.^38^ An intersectional approach is therefore essential in understanding health systems, and the dynamic and complex human experiences and interactions that make up those health systems.^26^ This approach would allow for better identification of the populations neglected or oppressed by the health system, and deliver interventions targeted to mitigate health inequities. This transition towards more equitable health systems and health outcomes can be strengthened by intersectional gender-transformative workforce policies.^49^ Examples of key attributes of a gender equitable health system are identified by Percival et al in their 2018 study, including provision of care for men and women across the life span; ensuring equitable access that is unrestricted by social, geographic and financial barriers; operating through evidence informed policies based on relevant, sex disaggregated health data; creation of equitable career opportunities for men and women health professionals; and ensuring equitable health outcomes among men and women and across age groups.^38^

### Increasing Women’s Representation Within Health System Leadership: Moving Beyond Gender Parity

The WHO Commission on Social Determinants of Health has clearly stated that social inequities, including gender inequity, are among causes of health disparities and recommends that governments strengthen political and legal systems to acknowledge and support marginalized groups to empower them to represent their needs and claim their rights.^15^ Commission on Social Determinants of Health recommends empowerment of women and marginalized groups at the micro-level of individual people, as well as ensuring their representation at the macro and meso decision-making levels within and beyond the health system to reduce health disadvantages that result from social inequities.^15^ Given women’s under-representation in leadership, we have focused on this issue in the present paper, while appreciating that gender equity goes beyond women’s rights. We have complemented the studies reviewed here by including a few studies on the experiences of men in the workforce to give additional context on how complex systems of oppression intersect with one another to create unique experiences of disadvantage.

Increasing women’s leadership in health systems at global, national and subnational levels is a vital step towards addressing women’s health challenges, and empowering and recognizing the majority of the health sector’s workforce. According to Downs et al, randomized trials have demonstrated that women in leadership positions of governmental organizations are more likely than men to implement policies that are supportive of women and children.^50^ Including diverse groups of women at all levels of health system leadership is important to ensuring that diverse experiences and perspectives are represented in health system decision-making and contribute to wider societal transformation.^51,52^ Newman argues that the positive effects of equal opportunity and gender equality in the health sector include: an increased health worker pipeline, an equal chance of being hired, being fairly paid and enjoying advancement opportunities, better work life balance, and improved health services.^22^ Improving women’s representation within health system leadership is therefore, beneficial to all.^52^

The first step towards identifying and implementing solutions in relation to women’s lack of representation within health system leadership is having a clear, robust understanding of the underlying obstacles to the participation of women, from all types of different social identities. Patriarchal structures manifest in complex, multifaceted and reinforcing ways. How these processes affect women of color, Indigenous women, women from LMICs, transgender and other groups of women and even men of color is underexplored and should be a priority for health systems research.^39^

### Where Do We Go From Here?

Based on the themes and intersecting identities emerging from the findings of this study and recommendations made in some of the reviewed papers, we propose the following overarching approaches.

To have more equitable, gender-responsive, and inclusive health systems that reflect these values at all hierarchical levels, examinations of gender biases in health system leadership, using intersectional analysis, is needed. An evidence-based understanding of the key factors influencing gender differences in leadership, and their impact, should contribute to policies and interventions that address drivers of inequity. Implicit and explicit biases and stereotypes are the heart of sexist and patriarchal systems and institutions. Such deeply held beliefs often work in insidious ways to exclude women from enjoying certain privileges and opportunities. Due to their implicit nature, rooted in centuries of patriarchal systems, acknowledgement and transformation of these biases requires conscious, constant effort done at the individual and societal level. Despite their prevalence, gender biases are not static nor universal, but actively debated, discussed and adapted at the individual level. These individual attempts must be collectively and systematically expanded through improved policies and programs in health systems to be more effective. Institutions should publicly state their commitment to gender equality and inform policies that tackle power and privilege imbalances, such as workplace gender equality policies and workplace inclusion and diversity policies.^12^

Furthermore, many studies pointed to the importance of mentorship in the career progression of women. Mentorship can provide professional support and guidance on career advancement, work-life balance, and professional resilience.^50^ Navigating complex workplaces or conditions such as parenthood can be done more easily when a mentor shares similar experiences and tips on best practices. Moreover, mentors can champion their proteges, advocate and create opportunities for them.^51^

The pervasive global gender stereotype that assigns women the role of primary caregiver for children is a significant limiting factor for women’s full participation in the workforce and their ascent to higher positions. This stereotype is further reinforced by cultural norms as observed in the studies done in Kenya, Lebanon, and Saudi Arabia. Women’s participation in health systems as well as their childcare responsibilities leaves them with little time to invest in their own health and well-being or to pursue leadership training or other resources that enable them to advance in their career. In the absence of institutional support, women overextend themselves to balance their personal and professional lives, plan pregnancies and manage childcare and develop professionally. This division of responsibilities can be improved by adoption of institutional gender-responsive policies that promote parental leave for both parents, flexible working hours, and family-friendly policies to ensure women can enjoy flexible career trajectories and have equal resources and access to leadership roles within health systems. Such policies promote shared parenthood, subsidized childcare and preserve women’s connection to their job during their childbearing years. Studies have proven that in fact having more women in leadership positions promotes adoption and implementation of these gender-responsive policies.^53,54^

#### Gender and Professional Cadre

Even though the health workforce consists of diverse cadres, medical doctors have historically been appointed to leadership positions. This has undermined the key role of other cadres to a functioning, well-balanced workforce. Leadership quotas for different cadres, management training for any health worker promoted to a leadership position, and mentorship provided by experienced members of staff can ensure a more equitable and inclusive leadership trajectory.

#### Gender and Race/Ethnicity

Sexism and racism both manifest implicitly and explicitly. Individuals at the intersection of these structures of exclusion are often overlooked. One solution to tackle racism in health systems has been the adoption of affirmative action policies, as mentioned in the South African studies. While such policies ensure representation in numbers, it is imperative to make sure selections under affirmative policies take into account other identities such as gender or financial status. Furthermore, implementation of such policies and quotas risks tokenization or race fatigue, as explained by Thackwell et al.^19^ In order to avoid such views, transparency in selection criteria and meritocracy, even in the affirmative action process can be helpful.

#### Gender and Culture

Combatting stereotypes and biases is a complex and long-term endeavor. However, health systems and organizations can take important measures to mitigate the negative effects of such biases and actively foster a more inclusive organizational culture where such biases are consistently challenged and eliminated. Implementation of the institutional gender equality policies mentioned in previous sections, adoption of transparent selection and promotion criteria can help avoid cultural biases that exclude competent women, especially of younger age, from leadership opportunities. Supportive initiatives such as mentorship programs and training courses can further assist and invest in the existing health workforce. Mentors can be crucial in sharing experiences of navigating challenging workplaces or conditions and championing their younger colleagues in promotion decisions. Institutional support such as flexible working hours and paid parental leave can improve the working conditions of health workers with childcare responsibilities.

#### Gender and Financial Status

A recurring theme in the reviewed studies was the financial barriers women faced to pursue further education or leadership training. Investing in the professional development of all health workers, beyond their technical knowledge and expertise, and provision of scholarship schemes and loans for further education can be beneficial to the career progression of health workers. It is important to note that allocating a proportion of such funds to women health workers would ensure that women who are from lower financial backgrounds, those from cadres with lower income, those who have to prioritize personal funds for their children and family, and those who may be overburdened with professional and domestic responsibilities who may not even be prompted to apply for such funds, are not overlooked and ignored by the system. Mentors can be crucial in this step as well, identifying opportunities and nominating candidates.

Consistent implementation of the aforementioned recommendations requires a broader policy shift towards an intersectionality framework. Based on the conclusions drawn from reviewing the literature, we have identified overarching recommendations for policy-makers to describe, commit, act, and transform health systems through an intersectionality framework to reduce inequities. This can be achieved by the reflexivity of policy-makers that ensures recognition of the privilege of being in the decision-making position, and reveals harmful biases, assumptions, stereotypes and exclusions, subsequently leading to the inclusion of a diversity of perspectives. Being mindful and inclusive of, and responsive to, the needs of diverse groups of health workers, considering intersecting social locations and their interconnected domains of power can lead to moving beyond intersectionality-aware policies to intersectionality-transformative policies.^40,55,56,58–60^

### Study Limitations

This study was limited by only exploring articles published in English as the authors main language. We were further constrained by the fact that this project was part of a degree thesis project and therefore limited to one reviewer. Since the initial inclusion criteria included only studies which explicitly used intersectionality as a lens, articles which addressed different social stratifiers but without an intersectionality approach would not have been captured within the search strategy. While this narrowed the number of results, it also showed the dearth of evidence using an intersectionality framework in this field.

## Conclusion

Achieving gender equity in health systems leadership at all levels is fundamental to ensuring that the diversity of human resources in the global community is appreciated.^4^ If we are to advocate for reforming the workplace and workforce in health systems, and for equal opportunities in leadership positions in health systems across nations and in global health, it is imperative to move beyond gender and be cognizant of the different challenges that women face in their career advancement in different settings, due to the intersection of their gender with other social identities, and not dismiss these differences by oversimplifying gender as the only defining aspect of one’s identity. Since using an intersectionality framework in considering women’s leadership in health systems in LMICs is virtually absent from the discourse, incorporating an intersectional framework in addressing their participation in leadership and researching evidence around it can ensure a more holistic approach that does not only promote advancement of the stereotypically portrayed women in leadership, but all women from different nationalities and races, professional cadres, religions and economic backgrounds. As efforts to reduce gender inequity in health systems are gaining momentum, it is important to look beyond gender as an all-encompassing disadvantage and take into account other social identities that interact with gender and adopt this shift to an intersectional paradigm in programs and policies that are collectively amplified by the health sector. The result of this effort is not only more gender equity in the health workforce, but more equity in general, improving health systems’ functioning and downstream health benefits more broadly.

## Figures and Tables

**Figure:**
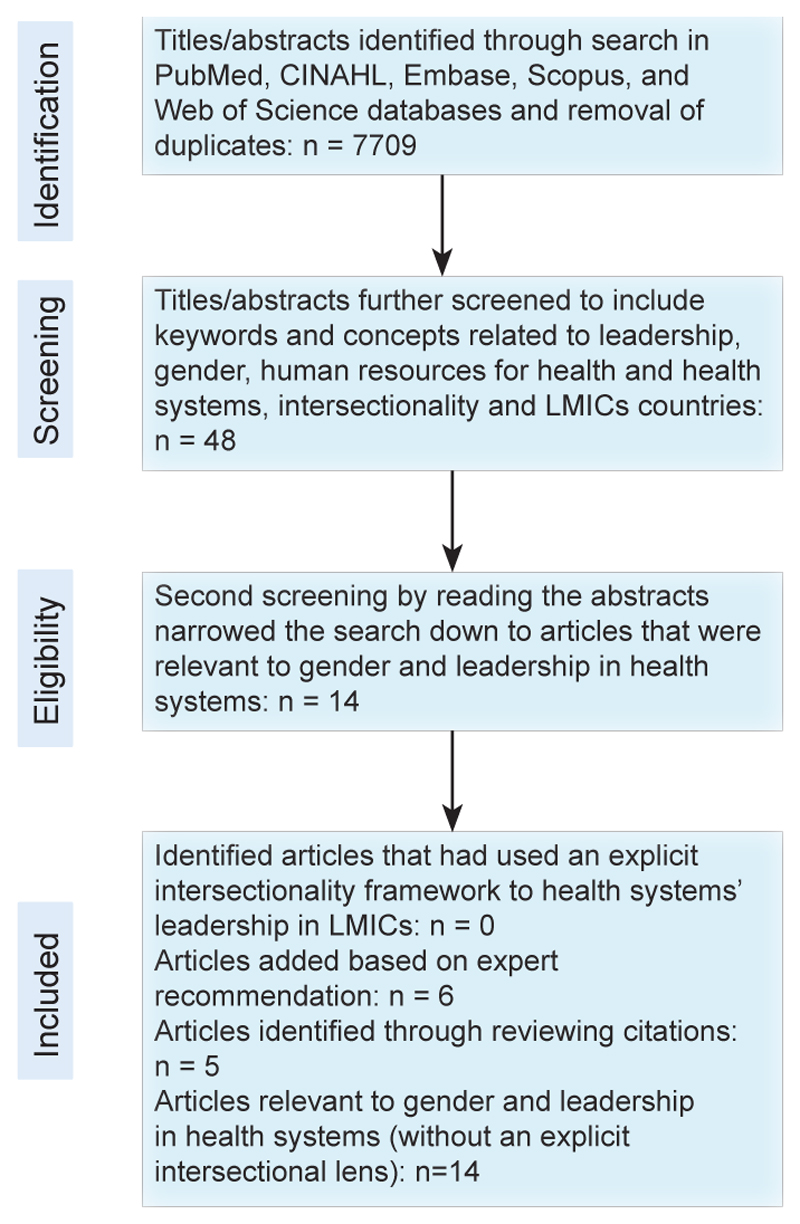
PRISMA Flow Chart. Abbreviations: PRISMA, Preferred Reporting Items for Systematic Reviews and Meta-Analyses; LMICs, low- and middle-income countries.

**Table 1:** Inclusion/Exclusion Criteria

Parameters	Inclusion Criteria	Exclusion Criteria
Language	Studies written in English	Studies not written in English
Time frame	Studies published from 2000 onwards	Studies published before 2000
Study type	Primary research, literature reviews	Grey literature, opinion pieces, commentary
Study focus	Intentionally uses intersectionality as a lens	Does not intentionally use intersectionality as a lens, meaning there was not a discussion about intersectionality as an approach, perspective, framework, or lens (this exclusion criterion was later abandoned as adhering to it would have yielded zero results)
	At least two social stratifiers included	Only one social stratifier included
	Specifically relates to leadership/leadership positions	Does not relate to leadership/leadership positions
	Relates to health systems and especially HRH	Does not relate to health systems
	Focuses on leadership within the health system	Does not focus on leadership within the health system
	Focuses on gender/gender inequity	Does not focus on gender/gender inequity

Abbreviation: HRH, human resources for health.

**Table 2:** Summary of Findings

Authors	Study	Social Stratifiers Included	Systems and Structures Involved
Dhatt et al^4^	The role of women’s leadership and gender equity in leadership and health system strengthening	Gender, Cadre, Race	Sexism, patriarchy, governance, professional elitism, racism
Hay et al^5^	Disrupting gender norms in health systems: making the case for change	Gender	Sexism, patriarchy
George^14^	Human resources for health: a gender analysis background paper	Gender	Sexism, patriarchy
Weber et al^16^	Gender norms and health: insights from global survey data	Gender	Sexism, patriarchy
Larson et al^17^	10 Best resources on... intersectionality with an emphasis on low- and middle-income countries	Gender, Race, Financial status	Sexism, patriarchy, racism
Thackwell et al^19^	Race trouble: experiences of Black medical specialist trainees in South Africa	Gender, Race	Sexism, patriarchy, racism, colonialism
van Rensburg^20^	South Africa’s protracted struggle for equal distribution and equitable access – still not there	Gender, Cadre, Race	Sexism, patriarchy, racism, colonization, professional elitism
Filby et al^21^	What prevents quality midwifery care? A Systematic mapping of barriers in low and middle income countries from the provider perspective	Gender, Cadre, Race	Sexism, patriarchy, professional elitism, racism
Newman^22^	Time to address gender discrimination and inequality in the health workforce	Gender, Age	Sexism, patriarchy, ageism
Muraya et al^25^	‘Gender is not even a side issue...it’s a non-issue’: career trajectories and experiences from the perspective of male and female healthcare managers in Kenya	Gender, Cadre, Race/ Ethnicity, Financial status	Sexism, patriarchy, governance, professional elitism, racism/ xenophobia
Shung-King et al^26^	Leadership experiences and practices of South African health managers: what is the influence of gender? - a qualitative, exploratory study	Gender, Cadre, Race	Sexism, patriarchy, governance, professional elitism, racism, historical forces and colonization
Tominc et al^27^	Perceived gender equality in managerial positions in organizations	Gender	Sexism, patriarchy
Acosta et al^28^	Achieving gender equity is our responsibility: leadership matters	Gender	Sexism, patriarchy
Vong et al^29^	Why are fewer women rising to the top? A life history gender analysis of Cambodia’s health workforce	Gender	Sexism, patriarchy, governance
Kodagoda^30^	Working long hours and its impact on family life: experiences of women professionals and managers in Sri Lanka	Gender	Sexism, patriarchy
Zeinali et al^31^	Intersectionality and global health leadership: parity is not enough	Gender, Cadre	Sexism, patriarchy, political institutions, governance, professional elitism
Alwazzan and Rees^32^	Women in medical education: views and experiences from the Kingdom of Saudi Arabia	Gender, Culture, Generation/Age, Religion	Sexism, patriarchy, ageism, religion
Ioannidou et al^33^	Empowering women researchers in the new century: IADR’s strategic direction	Gender	Sexism, patriarchy
Tlaiss^34^	Women in healthcare: barriers and enablers from a developing country perspective	Gender, Culture, Religion	Sexism, patriarchy, religion
Williams^35^	The Glass Escalator, Revisited	Gender, Cadre, Race	Sexism, patriarchy, racism, professional elitism
George^36^	Nurses, community health workers, and home carers: gendered human resources compensating for skewed health systems	Gender, Cadre, Race	Sexism, patriarchy, professional elitism, racism
Chua et al^37^	Social capital in Singapore: gender differences, ethnic hierarchies, and their intersection	Gender, Ethnicity	Sexism, ethnic discrimination, Historical factors
Percival et al^38^	Are health systems interventions gender blind? Examining health system reconstruction in conflict affected states	Gender	Sexism
Morgan et al^39^	The foundation and consequences of gender bias in grant peer review processes	Gender	Sexism, patriarchy
Gupta et al^40^	Gender equality and gender norms: framing the opportunities for health	Gender	Sexism, patriarchy

Abbreviation: IADR, International Association for Dental Research.
